# Development of β-carotene, lysine, and tryptophan-rich maize (*Zea mays*) inbreds through marker-assisted gene pyramiding

**DOI:** 10.1038/s41598-022-11585-y

**Published:** 2022-05-20

**Authors:** Neelima Chandrasekharan, Nagalakshmi Ramanathan, Bharathi Pukalenthy, Sarankumar Chandran, Dhasarathan Manickam, Karthikeyan Adhimoolam, Ganesan Kalipatty Nalliappan, Sudha Manickam, Ravikesavan Rajasekaran, Vellaikumar Sampathrajan, Vignesh Muthusamy, Firoz Hossain, Hari Shankar Gupta, Senthil Natesan

**Affiliations:** 1 Department of Plant Biotechnology, Centre for Plant Molecular Biology and Biotechnology, Tamil Nadu Agricultural University, Coimbatore, 641003 Tamil Nadu India; 2 Department of Plant Breeding and Genetics, Centre for Plant Breeding and Genetics, Tamil Nadu Agricultural University, Coimbatore, 641003 Tamil Nadu India; 3 Department of Plant Breeding and Genetics, Agricultural College and Research Institute, Tamil Nadu Agricultural University, Madurai, 625105 Tamil Nadu India; 4 Department of Plant Molecular Biology and Bioinformatics, Centre for Plant Molecular Biology and Biotechnology, Tamil Nadu Agricultural University, Coimbatore, 641003 Tamil Nadu India; 5 Department of Biotechnology, Agricultural College and Research Institute, Tamil Nadu Agricultural University, Madurai, 625105 Tamil Nadu India; 6 Department of Forage Crops, Centre for Plant Breeding and Genetics, Tamil Nadu Agricultural University, Coimbatore, 641003 Tamil Nadu India; 7 Department of Millets, Center for Plant Breeding and Genetics, Tamil Nadu Agricultural University, Coimbatore, 641003 Tamil Nadu India; 8 Division of Genetics, Indian Agricultural Research Institute, New Delhi, India

**Keywords:** Biotechnology, Molecular biology, Plant sciences

## Abstract

Maize (*Zea mays* L.) is the leading cereal crop and staple food in many parts of the world. This study aims to develop nutrient-rich maize genotypes by incorporating *crtRB1* and *o2* genes associated with increased β-carotene, lysine, and tryptophan levels. UMI1200 and UMI1230, high quality maize inbreds, are well-adapted to tropical and semi-arid regions in India. However, they are deficient in β-carotene, lysine, and tryptophan. We used the concurrent stepwise transfer of genes by marker-assisted backcross breeding (MABB) scheme to introgress *crtRB1* and *o2* genes. In each generation (from F_1_, BC_1_F_1_–BC_3_F_1_, and ICF_1_–ICF_3_), foreground and background selections were carried out using gene-linked (*crtRB1* 3′TE and umc1066) and genome-wide simple sequence repeats (SSR) markers. Four independent BC_3_F_1_ lines of UMI1200 × CE477 (Cross-1), UMI1200 × VQL1 (Cross-2), UMI1230 × CE477 (Cross-3), and UMI1230 × VQL1 (Cross-4) having *crtRB1* and *o2* genes and 87.45–88.41% of recurrent parent genome recovery (RPGR) were intercrossed to generate the ICF_1_-ICF_3_ generations. Further, these gene pyramided lines were examined for agronomic performance and the β-carotene, lysine, and tryptophan contents. Six ICF_3_ lines (DBT-IC-β_1_σ_4_-4-8-8, DBT-IC-β_1_σ_4_-9-21-21, DBT-IC-β_1_σ_4_-10-1-1, DBT-IC-β_2_σ_5_-9-51-51, DBT-IC-β_2_σ_5_-9-52-52 and DBT-IC-β_2_σ_5_-9-53-53) possessing *crtRB1* and *o2* genes showed better agronomic performance (77.78–99.31% for DBT-IC-β_1_σ_4_ population and 85.71–99.51% for DBT-IC-β_2_σ_5_ population) like the recurrent parents and β-carotene (14.21–14.35 μg/g for DBT-IC-β_1_σ_4_ and 13.28–13.62 μg/g for DBT-IC-β_2_σ_5_), lysine (0.31–0.33% for DBT-IC-β_1_σ_4_ and 0.31–0.34% for DBT-IC-β_2_σ_5_), and tryptophan (0.079–0.082% for DBT-IC-β_1_σ_4_ and 0.078–0.083% for DBT-IC-β_2_σ_5_) levels on par with that of the donor parents. In the future, these improved lines could be developed as a cultivar for various agro-climatic zones and also as good genetic materials for maize nutritional breeding programs.

## Introduction

Maize, an important cereal, is life for millions in the global population, as a source of protein, vitamins, minerals, oils, and dietary fibre. The crop is cultivated widely in diverse agroecology across the globe and has the highest genetic yield potential among the cereals. It is grown in more than 160 countries with a total production of 1.05 million thousand tonnes and 28.90 million tonnes in India for the year 2019^1^. Maize is a rich source of provitamin A and non-provitamin A carotenoids. The carotenoids are synthesized in the maize endosperm via the carotenoid biosynthesis pathway that originates from the isoprenoid precursor, geranyl pyrophosphate, supplied by the MEP pathway^2^. Through a series of enzyme-mediated reactions, phytoene, the first carotenoid compound, is synthesized and enzymatically converted to lycopene. This is the branch point of the pathway, and further conversion depends on the cyclization of the lycopene molecule. An asymmetric cyclization would produce an α-carotene molecule, and a symmetric cyclization would yield a β-carotene molecule, forming the provitamin A carotenoids in maize^3^.

Among the provitamin A carotenoids, β-carotene has the highest provitamin A potential due to the presence of two β-ionone rings. β-carotene is further hydroxylated to produce β-cryptoxanthin and further to zeaxanthin and ABA which are non-provitamin A carotenoids^4^. Hence, in normal maize, due to the conversion of β-carotene to non-provitamin A carotenoids, a micronutrient deficiency occurs, particularly the vitamin A deficiency (VAD). Maize is also a staple food in many of the sub-Saharan and Latin American countries, and hence, VAD would pose an important threat to the population, specifically the pregnant women and infants, resulting in complications such as blindness and growth retardation^5,6^. In 2018, a study conducted by UNICEF revealed that children aged between 6 and 59 months from East Asia and the Pacific regions received the highest two-dosage of vitamin A supplements with 75% from the African countries and 59% from the South Asian countries^7^. Therefore, there is a pressing need for alleviating this micronutrient complication, and since the carotenoid compounds are naturally accumulated in the edible part of the maize endosperm, it becomes an ideal crop for biofortification.

Several studies have identified various genes that are directly involved in the variation of the β-carotene pathway by directly or indirectly modifying the carotene biosynthesis pathway. The *LcyE* and the *crtRB1* genes were shown to be directly involved in influencing the beta carotene levels in the maize endosperm^8,9^. The precise manipulation of the *crtRB1* gene has shown to favorably increase the beta carotene concentration in previous studies^10,11^. Yan et al. identified the *crtRB1* gene responsible for this conversion and also three polymorphisms that influence the variation in the carotenoid concentration. The polymorphism in the 3’TE region with the favorable allele (543 bp) increases the carotene concentration in maize^9–11^.

Maize also contains two protein fractions viz*.,* zein and non-zein, where zein proteins are predominant. However, these zein proteins lack essential amino acids like lysine and tryptophan and hence induce Protein Energy Malnutrition (PEM). Several natural mutants (i.e., *opaque* 2 (*o2*)^12^, *floury 2* (*fl2*)^13^, *opaque 7 (o7)*^14^*, opaque 6 (o6)*^15^*, floury 3 (fl3)*^14^*)* have shown to increase these essential amino acids in maize of which *o2* has been widely studied. The *o2* mutant is known to decrease the zein fraction and increase the non-zein fraction which is naturally high in essential amino acids^16–18^. The large genetic variation present in maize makes it an ideal crop for nutritional improvement specifically in regard to micronutrient deficiencies. Marker-assisted backcross breeding (MABB) has been shown to be a promising technique to introgress several nutritionally important genes in many crops including maize^19^. Nutritional traits viz., provitamin A, higher protein content, high Zn, Fe, and Se content have been improved in maize through the MABB technique^8,20–23^.

Several studies in India and other parts of the world have successfully introgressed either *crtRB1* or *o2* into popular elite lines and improved the β-carotene, lysine, and tryptophan contents^24–30^. The common determinant in all the previous studies is the introgression of a single factor into an established variety. By adopting the technique of gene pyramiding, varieties can be produced with broad sense capabilities and essentially more important genetic stocks. Especially by bringing improved versions of β-carotene, lysine, and tryptophan into a single genotype, the time required to improve the plants individually is reduced and would also provide a superior genotype with several favourable nutritional traits. This has now become possible due to the advances made in technology as well as the identification of new molecular markers and integrated techniques developed for efficient selection^26,28,31–34^. Considering these, this study is planned to develop an intercross population and pyramid the *crtRB1* and *o2* simultaneously in the background of elite genotypes.

## Results

### Transfer of *crtRB1* and *o2* genes into UMI1200

A total of 27 and 23 F_1_s were produced in cross-1 and cross-2, and their heterozygosity was confirmed via foreground markers associated with *crtRB1* and *o2* genes. The healthy F_1_s from both crosses were backcrossed with a recurrent parent to produce 106 and 232 BC_1_F_1_ lines, and again heterozygous conditions were confirmed in BC_1_F_1_ lines using foreground markers. All the heterozygous positives were subjected to background selection with 112 and 106 polymorphic markers. They showed 52.82–56.41% and 62.13–74.25% of RPGR with an average of 54.84% and 69.38% in cross-1 and cross-2. Further, one BC_1_F_1_ line from each cross with *crtRB1* and *o2* genes and maximum RPGR was selected and backcrossed with a recurrent parent to produce 136 and 218 BC_2_F_1_ lines. Following similar selection procedures, BC_2_F_1_ generation was advanced to BC_3_F_1_. A total of 85 and 109 BC_3_F_1_ lines were produced to the cross-1 and cross-2, and foreground selection revealed that the 24 and 31 BC_3_F_1_ lines had *crtRB1* and *o2* genes in the heterozygous condition. All these plants were subjected to background selection, and they exhibited 86.35–88.52% and 86.14–88.21% of RPGR with an average of 87.74 and 87.45% (Supplementary Tables S1, S2). Among them, two lines, DBT 1-1-1-17-5-14 from cross-1 and DBT 4-1-1-10-10-16 from cross-2 having maximum RPGR, were used to develop the intercross population (designated as DBT-IC-β_1_σ_4_).

### Transfer of *crtRB1* and *o2* genes into UMI1230

With the support of foreground markers, *crtRB1* and *o2* genes heterozygous lines were confirmed in F_1_s from cross-3 and cross-4. The F_1_s were backcrossed with a recurrent parent to produce 121 and 160 BC_1_F_1_ lines. Among them, 42 and 68 BC_1_F_1_ lines possessing *crtRB1* and *o2* genes in their heterozygous condition were identified in cross-3 and cross-4 using foreground markers and were subjected to background selection with 114 and 90 polymorphic SSR markers. Background selection revealed 53.87–57.69% and 68.60–76.20% of RPGR with an average of 55.12% and 72.70%. One BC_1_F_1_ line from each cross possessing *crtRB1* and *o2* genes and maximum RPGR was selected and backcrossed with a recurrent parent to produce 146 and 153 BC_2_F_1_ lines. Applying the same strategy, 5 BC_3_F_1_ and 10 BC_3_F_1_ lines possessing *crtRB1* and *o2* genes and maximum RPGR were identified. The BC_3_F_1_ lines from cross-3 and cross-4 exhibited 86.75–88.84% and 87.56–89.42% of RPGR with an average of 87.84% and 88.41% (Supplementary Tables S1, S2). The two BC_3_F_1_ lines, (DBT 2-1-4-7-1-9) and (DBT 5-1-14-5-8-7) from cross-3 and 4 having maximum RPGR, were used to develop the intercross population (designated as DBT-IC-β_2_σ_5_).

### Stacking of *crtRB1* and *o2* genes

**Figure 1:**
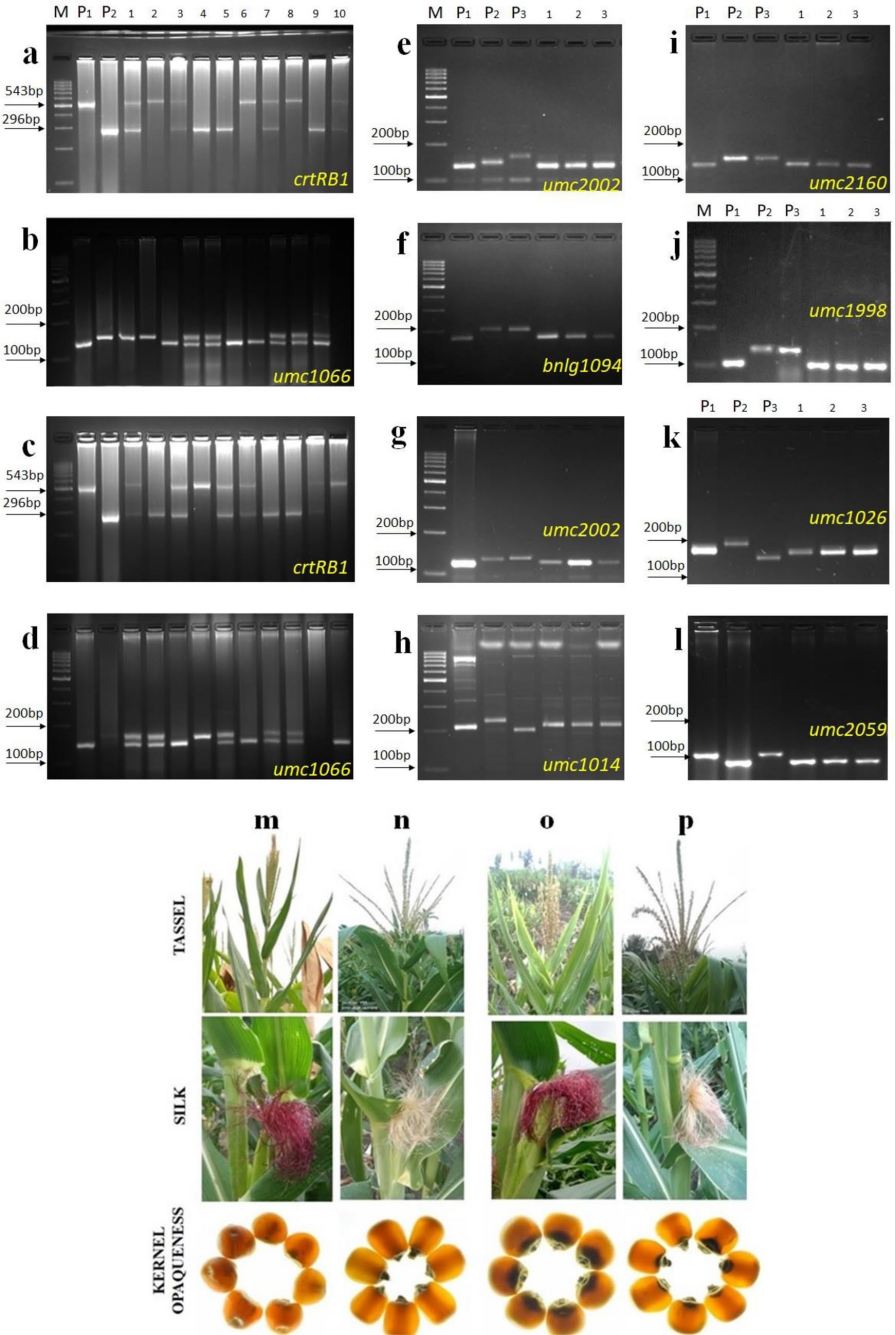
Foreground and background selection and morphological traits evaluation in ICF_2_ and ICF_3_ populations. (**a**) Foreground selection of ICF_2_ lines from DBT-IC-β_1_σ_4_ using *crtRB1* gene specific marker *crtRB1* 3’TE, (M) Ladder (100 bp), (P_1_) CE477, (P_2_) UMI1200, (1–10) ICF_2_ plants; (**b**) Foreground selection of ICF_2_ lines from DBT-IC-β_1_σ_4_, using *o2* gene linked marker umc1066, (M) Ladder (100 bp), (P_1_) UMI1200, (P_2_) VQL 1, (1–10) ICF_2_ plants; (**c**) Foreground selection of ICF_2_ lines from DBT-IC-β_2_σ_5_ using *crtRB1* gene specific marker *crtRB1* 3’TE, (M) Ladder (100 bp), (P_1_) CE477, (P_2_) UMI1230, (1–10) ICF_2_ plants; (**d**), Foreground selection of ICF_2_ lines from DBT-IC-β_2_σ_5_ using *o2* gene linked marker umc1066, (M) Ladder (100 bp), (P_1_) UMI1230, (P_2_) VQL 1, (1–10) ICF_2_ plants; (**e**, **f**, **i** and **j**), Background selection of ICF_3_ lines from DBT-IC-β_1_σ_4_, (M) Ladder (100 bp), (P_1_) UMI1200, (P_2_) CE477, (P_3_) VQL 1, (1–3) ICF_3_ plants; (**g**, **h**, **k** and **l**), Background selection of ICF_3_ lines from DBT-IC-β_2_σ_5,_ (M) Ladder (100 bp), (P_1_) UMI1230, (P_2_) CE477, (P_3_) VQL 1, (1–3) ICF_2_ plants; (**m**–**p**), Evaluation of morphological traits in ICF_3_ lines. UMI 1200 (**m**), UMI1230 (**n**), DBT-ICβ_1_σ_4_-4-8-8 (**o**), DBT-IC-β_2_σ_5_-9-53-53 (**p**).

The line DBT 1-1-1-17-5-14 (derived from cross 1) was used as the female parent and DBT 4-1-1-10-10-16 (derived from cross 2) as the male parent in the development of intercross population (DBT-IC-β_1_σ_4_) to pyramid *crtRB1* and *o2* genes. Among the 128 ICF_1_ lines, 64 lines were confirmed to be heterozygous for two target genes. Of these, 64 ICF_1_ were selected and selfed to obtain 40 ICF_2_ lines. Foreground selection was conducted in ICF_2_ lines to trace the lines carrying a combination of two genes. Based on foreground selection and the phenotyping of kernels for opaqueness (25%), a total of 9 homozygous lines with *crtRB1* and *o2* genes were identified. Chi-square test on the 9 lines revealed that the population followed the expected Mendelian ratio of 1:2:1 (Table 1; Fig. 1). Background selection was done in those selected 9 lines with 148 polymorphic SSR markers and selfed to produce ICF_3_ generation (Supplementary Table S3). Eventually, ICF_3_ lines, (DBT-IC-β_1_σ_4_-4-8-8, DBT-IC-β_1_σ_4_-9-21-21, and DBT-IC-β_1_σ_4_-10-1-1 having 90.47%, 90.62%, and 89.54% of RPGR with 25% opaqueness, were developed. Likewise, to pyramid the *crtRB1* and *o2* genes, another intercross population (DBT-IC-β_2_σ_5_) was generated using the line DBT 2-1-4-7-1-9 (Cross 3) as the female parent and DBT 5-1-14-5-8-7 (Cross 4) as the male parent. Foreground markers confirmed the heterozygous form of *crtRB1* and *o2* genes in ICF_1_ lines. Then, 72 healthy ICF_1_ lines were selfed to produce 45 ICF_2_ lines. Foreground selection coupled with phenotyping of kernels for opaqueness resulted in 9 homozygous ICF_2_ lines with *crtRB1* and *o2* genes. All these lines were subjected to background selection and then selfed to produce ICF_3_ generation. Finally, 3 lines, DBT-IC-β_2_σ_5_-9-51-51, DBT-IC-β_2_σ_5_-9-52-52, and DBT-IC-β_2_σ_5_-9-53-53 having 91.71%, 89.05%, and 88.14% RPGR and opaqueness of 25% were generated (Supplementary Table S3; Fig. 1). Collectively, 6 ICF_3_ lines DBT-IC-β_1_σ_4_-4-8-8, DBT-IC-β_1_σ_4_-9-21-21, DBT-IC-β_1_σ_4_-10-1-1, DBT-IC-β_2_σ_5_-9-51-51, DBT-IC-β_2_σ_5_-9-52-52, and DBT-IC-β_2_σ_5_-9-53-53 containing *crtRB1* and *o2* genes were developed with 25% opaqueness.

**Table 1:** Segregation pattern of *o2* and *crtRB1* allele in intercross (IC_2_) population of DBT-IC-β_1_σ_4_ and DBT-IC-β_2_σ_5_.

*o2*							*crtRB1*					
Ear	Total number of plants genotyped	Genotypic class			χ^2^	P-value	Total number of plants genotyped	Genotypic class			χ^2^	P value
		*O2O2*	*O2o2*	*o2o2*				Allele3	Allele3/Allele1	Allele1		
**DBT-IC-β**_**1**_**σ**_**4**_												
DBT-IC-β_1_σ_4_-4-1	110	27	58	25	0.400 ns	0.819	110	28	58	24	0.618 ns	0.734
DBT-IC-β_1_σ_4_-4-3	95	25	49	21	0.432 ns	0.806	95	25	50	20	0.789 ns	0.674
DBT-IC-β_1_σ_4_-4-6	116	30	57	29	0.052 ns	0.974	116	31	60	25	0.759 ns	0.684
DBT-IC-β_1_σ_4_-4-8	115	28	60	27	0.235 ns	0.889	115	31	58	26	0.443 ns	0.801
DBT-IC-β_1_σ_4_-9-19	92	23	50	19	1.043 ns	0.593	92	25	50	19	1.217 ns	0.544
DBT-IC-β_1_σ_4_-9-21	121	30	62	29	0.091 ns	0.956	121	32	63	26	0.802 ns	0.670
DBT-IC-β_1_σ_4_-9-23	105	28	55	22	0.924 ns	0.630	105	28	55	22	0.924 ns	0.630
DBT-IC-β_1_σ_4_-10-1	102	27	53	22	0.647 ns	0.724	102	27	51	24	0.176 ns	0.916
DBT-IC-β_1_σ_4_-10-4	94	26	47	21	0.532 ns	0.766	94	24	50	20	0.723 ns	0.696
**DBT-IC-β**_**2**_**σ**_**5**_												
DBT-IC-β_2_σ_5_-9-25	94	25	49	20	0.702 ns	0.704	94	23	51	20	0.872 ns	0.647
DBT-IC-β_2_σ_5_-9-34	112	30	58	24	0.786 ns	0.675	112	29	60	23	1.214 ns	0.545
DBT-IC-β_2_σ_5_-9-42	95	25	50	20	0.789 ns	0.674	95	25	49	21	0.432 ns	0.806
DBT-IC-β_2_σ_5_-9-45	106	28	55	23	0.623 ns	0.732	106	27	55	24	0.321 ns	0.852
DBT-IC-β_2_σ_5_-9-49	100	25	52	23	0.240 ns	0.887	100	26	53	21	0.860 ns	0.651
DBT-IC-β_2_σ_5_-9-50	97	25	51	21	0.588 ns	0.745	97	25	52	20	1.021 ns	0.600
DBT-IC-β_2_σ_5_-9-51	97	27	49	21	0.753 ns	0.686	97	22	54	21	1.268 ns	0.530
DBT-IC-β_2_σ_5_-9-52	103	25	55	23	0.553 ns	0.758	103	28	53	22	0.786 ns	0.675
DBT-IC-β_2_σ_5_-9-53	94	25	49	20	0.702 ns	0.704	94	24	49	21	0.362 ns	0.835

*O2O2,* Homozygous dominant; *O2o2*, Heterozygotes; *o2o2*, Homozygous recessive (Favourable); Allele 3 (Unfavourable); Allele 3/1 (Unfavourable); Allele 1 (Favourable); ns (Non significant).

### Evaluation of ICF_3_ generation for morphological traits

The newly developed 6 ICF_3_ line’s agronomical performance was evaluated (Fig. 1) by measuring 14 morphological traits and estimating the similarity percentage compared to the recurrent parent (Tables 2, 3). The three improved lines DBT-IC-β_1_σ_4_-4-8-8, DBT-IC-β_1_σ_4_-9-21-21, and DBT-IC-β_1_σ_4_-10-1-1 from the DBT-IC-β_1_σ_4_ population showed more than 90% similarity to the recurrent parent UMI1200 for most of the traits. The same was the case for the three improved lines from DBT-IC-β_2_σ_5_. In the DBT-IC-β_1_σ_4_ population, the similarity percentage ranged from 77.78% (NTB) to 99.31% (LB), and DBT-IC-β_1_σ_4_-4-8-8 showed the highest similarity percentage of 99.31% for LB, followed by DBT-IC-β_1_σ_4_-10-1-1 showing a similarity percentage of 99.28% for EW. In the DBT-IC-β_2_σ_5_ population, the similarity percentage ranged from 85.71% (NTB and NKRE) to 99.51% (LL), and DBT-IC-β_2_σ_5_-9-53-53 had the highest similarity percentage of 99.51% (LL), followed by DBT-IC-β_2_σ_5_-9-52-52 having a similarity percent of 98.86% for EL.

**Table 2:** Comparison of the double positive lines in the ICF_3_ generation of DBT-IC-β_1_σ_4_ along with its recurrent parents for the recovery percentage of morphological traits.

Morphological traits	Recurrent parent	Identified positive lines			Recovery percentage (%)		
DBT-IC-β_1_σ_4_	UMI1200	DBT-IC-β_1_σ_4_-4-8-8	DBT-IC-β_1_σ_4_-9-21-21	DBT-IC-β_1_σ_4_-10-1-1	DBT-IC-β_1_σ_4_-4-8-8	DBT-IC-β_1_σ_4_-9-21-21	DBT-IC-β_1_σ_4_-10-1-1
Days to tasseling (days)	58.00	56.00	55.00	57.00	96.55	94.83	98.28
Days to silking (days)	60.00	59.00	58.00	60.00	98.33	96.67	98.33
Plant height (cm)	155.87	154.68	152.89	153.00	99.24	98.09	98.16
Ear height (cm)	76.84	73.85	67.75	72.02	96.11	88.17	93.73
Tassel length (cm)	21.34	20.42	21.08	19.68	95.69	98.78	92.22
Number of tassel branches	9.00	7.00	7.00	8.00	77.78	77.78	88.89
Leaf length (cm)	57.24	56.66	55.31	54.53	98.99	96.63	95.27
Leaf breadth (cm)	7.20	7.15	6.87	7.06	99.31	95.42	98.06
Ear length (cm)	15.60	14.40	14.80	14.50	92.31	94.87	92.95
Number of kernel rows per ear	12.00	10.00	10.00	10.00	83.33	83.33	83.33
Number of kernels per row	26.00	23.00	24.00	24.00	88.46	92.31	92.31
Ear weight (g)	121.47	120.20	119.90	120.60	98.95	98.71	99.28
100 kernel weight (g)	28.76	26.50	27.20	25.80	92.14	94.58	89.71
Single plant yield	99.98	96.71	98.31	95.86	96.73	98.33	95.88

**Table 3:** Comparison of the double positive lines in the ICF_3_ generation of DBT-IC-β_2_σ_5_ along with its recurrent parents for the recovery percentage of morphological traits.

Morphological traits	Recurrent parent	Identified positive lines			Recovery percentage (%)		
DBT-IC-β_2_σ_5_	UMI1230	DBT-IC-β_2_σ_5_-9-51-51	DBT-IC-β_2_σ_5_-9-52-52	DBT-IC-β_2_σ_5_-9-53-53	DBT-IC-β_2_σ_5_-9-51-51	DBT-IC-β_2_σ_5_-9-52-52	DBT-IC-β_2_σ_5_-9-53-53
Days to tasseling (days)	60.00	57.00	58.00	59.00	95.00	96.67	98.33
Days to silking (days)	62.00	59.00	60.00	61.00	95.16	96.77	98.39
Plant height (cm)	158.40	156.28	153.94	150.25	98.66	97.18	94.85
Ear height (cm)	81.00	79.06	77.75	76.57	97.60	95.99	94.53
Tassel length (cm)	31.30	29.92	30.70	29.18	95.59	98.08	93.23
Number of tassel branches	14.00	12.00	13.00	12.00	85.71	92.86	85.71
Leaf length (cm)	63.20	58.19	62.11	62.89	92.07	98.28	99.51
Leaf breadth (cm)	7.50	7.20	7.10	7.20	96.00	94.67	96.00
Ear length (cm)	17.50	17.20	17.30	17.00	98.29	98.86	97.14
Number of kernel rows per ear	14.00	12.00	12.00	12.00	85.71	85.71	85.71
Number of kernels per row	25.00	24.00	22.00	24.00	96.00	88.00	96.00
Ear weight (g)	106.90	104.56	103.70	102.20	97.81	97.01	95.60
100 kernel weight (g)	25.20	22.12	23.21	24.11	87.78	92.10	95.67
Single plant yield	75.51	72.56	73.74	71.11	96.09	97.66	94.17

### β-carotene, lysine, and tryptophan contents in ICF_3_ lines

The β-carotene content in the recurrent parents, UMI1200 and UMI1230, was found to be 0.60 μg/g and 1.20 μg/g respectively, whereas, for the donor parent, CE477, the β-carotene content was found to be 15.20 μg/g. In the DBT-IC-β_1_σ_4_ population, the highest β-carotene content was found in DBT-IC-β_1_σ_4_-9-21-21 (14.35 μg/g) followed by DBT-IC-β_1_σ_4_-10-1-1 (14.29 μg/g) and DBT-IC-β_1_σ_4_-4-8-8 (14.21 μg/g). For the DBT-IC-β_2_σ_5_ population, DBT-IC-β_2_σ_5_-9-53-53 was found to contain the highest β-carotene content (13.62 μg/g) followed by DBT-IC-β_2_σ_5_-9-51-51 (13.54 μg/g) and DBT-IC-β_2_σ_5_-9-52-52 (13.28 μg/g). The lysine and tryptophan levels of the recurrent parents, UMI1200 and UMI1230, were found to be 0.26%, 0.013% and 0.25%, 0.020% respectively. The lysine and tryptophan levels of the donor parent VQL1 were found to be 0.42% and 0.087%, respectively. In the DBT-IC-β_1_σ_4_ population, DBT-IC-β_1_σ_4_-4-8-8 recorded the highest level of lysine and tryptophan (0.33% and 0.082%), which is comparable with that of the donor parent. This was followed by DBT-IC-β_1_σ_4_-10-1-1 (0.32% and 0.081%) and DBT-IC-β_1_σ_4_-9-21-21 (0.31% and 0.079%). For the DBT-IC-β_2_σ_5_ population, DBT-IC-β_2_σ_5_-9-51-51 was found to contain the highest levels of lysine and tryptophan (0.34% and 0.083%). This was followed by DBT-IC-β_2_σ_5_-9-52-52 (0.32% and 0.081%) and DBT-IC-β_2_σ_5_-9-53-53 (0.31% and 0.078%). The average lysine and tryptophan levels for the improved lines were 0.32% and 0.080% (Table 4).

**Table 4:** Lysine, tryptophan, and β carotene levels of the ICF_3_ improved double positive lines of DBT-IC-β_1_σ_4_ and DBT-IC-β_2_σ_5_.

Trait	UMI1200	UMI1230	CE477	VQL	DBT-IC-β_1_σ_4_			DBT-IC-β_2_σ_5_		
					DBT-IC-β_1_σ_4_-4-8-8	DBT-IC-β_1_σ_4_-9-21-21	DBT-IC-β_1_σ_4_-10-1-1	DBT-IC-β_2_σ_5_-9-51-51	DBT-IC-β_2_σ_5_-9-52-52	DBT-IC-β_2_σ_5_-9-53-53
β-Carotene	0.60	1.20	15.20	0.7	14.21	14.35	14.29	13.54	13.28	13.62
Lysine	0.26	0.25	0.13	0.42	0.33	0.31	0.32	0.34	0.32	0.31
Tryptophan	0.013	0.020	0.021	0.087	0.082	0.079	0.081	0.083	0.081	0.078

## Discussion

To improve maize lines with β-carotene, lysine, and tryptophan, in our study, we were able to pyramid two nutritionally important genes *crtRB1* and *o2* into a single genotype by way of intercrossing. In our breeding programme, four independent crosses (UMI1200 × CE477, UMI1200 × VQL1, UMI1230 × CE477, and UMI1230 × VQL1) were formed to incorporate the *crtRB1* and *o2* genes into two elite inbred lines UMI1200 and UMI1230. For the marker assisted backcrossing, selection using *crtRB1* gene-specific and umc1066 markers was done to identify four BC_3_F_1_ lines from each cross having the desired genes and maximum RPG%. The lines DBT 1-1-1-17-5-14 from cross-1 and DBT 4-1-1-10-10-16 from cross-2 were intercrossed to produce the DBT-IC-β_1_σ_4_ population, which in hindsight improved UMI1200 for β-carotene, lysine, and tryptophan levels. Similarly, the lines DBT 2-1-4-7-1-9 from cross-3 and DBT 5-1-14-5-8-7 from cross-4 were intercrossed to produce the DBT-IC-β_2_σ_5_ population, which improved UMI1230 for β-carotene lysine and tryptophan levels.

In all the IC generations (ICF_1_–ICF_3_), the same markers were used to ensure that the final products were double homozygotes for both the *crtRB1* and *o2* genes. In the ICF_2_ generation, generated lines from both DBT-IC-β_1_σ_4_ and DBT-IC-β_2_σ_5_ populations were subjected to the chi-square test. The results revealed that the population segregated in the expected Mendelian ratio of 1:2:1 without any significant distortion for both the markers. Similar results were also obtained by Veldboom and Lee.^35^ and Lu et al.^36^. The selected double positive lines were then used to produce the ICF_3_ generation wherein the double homozygotes were ensured using the crtRB1 3’TE gene-specific and umc1066 markers. In this way, we were able to stack the nutritionally important genes and develop lines that were improved for β-carotene, lysine, and tryptophan levels. Similar studies were reported by other researchers^26,28,31,33,34,37^. However, in our study, we were able to achieve gene stacking by intercrossing homogenous lines that already had enhanced levels of β-carotene, lysine, and tryptophan thereby reducing the breeding cycle due to which we were able to produce a homogenous population that was highly similar to that of the recurrent parent in a short amount of time. Moreover, we were able to improve UMI1200 and UMI1230 which are the parents of a popular maize hybrid CO6 that is most suited to the climatic regions of South India.

Recovery of recurrent parent genome was also achieved in both ICF_2_ and ICF_3_ generation using a total of 148 polymorphic SSR markers. A high RPG% was obtained in the ICF_2_ generation itself due to the initial improved lines used to produce the intercross population having low levels of unwanted linkage drag. Once the ICF_3_ generation was developed we were able to identify three lines in both cross combinations that were double homozygotes and had a high recovery of recurrent parent genome. These results are in accordance with earlier reports^19,26,32^. The analysis of the opaqueness in the ICF_2_ generation showed that all the seeds showed 25% opaqueness for both the cross combinations. This was achieved because the lines that were used to produce the intercross population were already established for the 25% opaqueness using the lightbox screening method. Therefore, the progenies of the ICF_3_ generation also showed only 25% opaqueness. These results are in accordance with the previous findings^24,29,33,38,39^.

Morphological trait evaluation in the ICF_3_ generation for both DBT-IC-β_1_σ_4_ and DBT-IC-β_2_σ_5_ populations revealed that the improved lines were having more than 90% similarity with that of the recurrent parent without any major differences in important yield characters like SPY and EW. It showed that complete recovery of important phenotypic and yield characters of the recurrent parent was attained in the pyramided lines along with the desired genes. The lines DBT-IC-β_1_σ_4_-10-1-1 and DBT-IC-β_1_σ_4_-9-21-21 from the DBT-IC-β_1_σ_4_ population and the lines DBT-IC-β_2_σ_5_-9-51-51 and DBT-IC-β_2_σ_5_-9-52-52 from the DBT-IC-β_2_σ_5_ population were found to have the highest similarity to the respective recurrent parents as far as the yield characters were concerned. Similar results were also reported by former researchers^28,34,40^.

The evaluation of nutritional contents proved that the ICF_3_ lines had improved levels of β-carotene, lysine, and tryptophan levels in comparison with their normal recurrent parents. In the DBT-IC-β_1_σ_4_ population, DBT-IC-β_1_σ_4_-9-21-21 and DBT-IC-β_1_σ_4_-4-8-8 had the highest levels of β-carotene, lysine, and tryptophan respectively. Whereas, in the DBT-IC-β_2_σ_5_ population, DBT-IC-β_2_σ_5_-9-51-51 and DBT-IC-β_2_σ_5_-9-53-53 had the highest levels of β-carotene, lysine, and tryptophan respectively. Similar results were also obtained by earlier studies^26,28,33,37^. The improved lines in both cross combinations obtained from the ICF_3_ generation not only have the target donor genes with elevated nutrition levels but also has the high recovery of recurrent parent genome as well as highest phenotypic similarity to that of the recurrent parents rendering them crucial genetic materials for further hybrid synthesis and other genetic studies.

The present study has resulted in the development of improved lines possessing two genes (*crtRB1* and *o2*) responsible for β-carotene, lysine, and tryptophan by marker-assisted gene pyramiding (MAGP) strategy. Thus, the pyramided inbred lines (UMI 1200 and UMI 1230) recorded a higher level of β-carotene, lysine, tryptophan, and better agronomic performance on par to donor parent and recurrent parents respectively. In the future, the promising improved lines could be developed as a cultivar for various agro-climatic zones and also as good genetic materials for maize nutritional breeding programs.

## Materials and methods

### Plant genetic materials

Maize inbreds, UMI1200, and UMI1230, well-adapted to tropical and semi-arid regions in India were selected as the recurrent parents. Because of their good combining ability, both were utilized to develop the CO6 hybrid. The inbreds seeds were obtained from the Department of Plant Genetic Resources, Centre for Plant Breeding and Genetics, Tamil Nadu Agricultural University, Coimbatore. VQL1 (Possessing *o2* associated with high lysine and tryptophan contents) and CE477 (Possessing *crtRB1* associated with high β-carotene content) were selected as donor parents. VQL1 was obtained from Vivekananda Parvatiya Krishi Anusandhan Sansthan (VPKAS), Almora, India, whereas CE477 was obtained from International Maize and Wheat Improvement Center, Mexico.

### Foreground and background selection

Foreground selection was done using closely linked markers to *crtRB1* and *o2* genes. The *crtRB1* gene located in chromosome 10 was selected using InDel marker *crtRB1* 3’TE^9^, whereas the *o2* gene located in chromosome 7 was selected using the simple sequence repeat (SSR) marker umc1066^41^. The background selection was done to examine the recurrent parent genome recovery (RPGR). It was performed using 248 SSR markers with known chromosomal positions distributing all ten maize chromosomes. All primer sequences were obtained from the maize genome database (www.maizegdb.org) and synthesized by Eurofins Ltd., Bangalore, India.

### DNA extraction and PCR amplification

Genomic DNA was isolated from a two-week-old plant following the method by Murray and Thompson^42^. The PCR analysis for the crtRB1 gene-specific marker *crtRB1* 3′TE (65F: ACACCACATGGACAAGTTCG) and (62R: ACACTCTGGCCCATGAACAC, 66R: ACAGCAATACAGGGGACCAG) was carried out in a 10 μl reaction containing 2 μl of 20 ng template DNA, 2 mM of MgCl_2_, 1 mM of dNTPs, 2 μM of primer pair and 1.5U of Taq polymerase. The screening followed the ‘touch down’ technique of an initial denaturation for 5 min at 94 °C, followed by 19 cycles of denaturation for 45 s at 94 °C, annealing for 30 s at 62 °C with a reduction of 0.5 °C in every cycle down to 54 °C and extension for 1 min at 72 °C followed by another 20 cycles of denaturation at 94 °C for 45 s, annealing at 54 °C for 30 s, extension at 72 °C for 1 min and 20 s and a final extension at 72 °C for 10 min. The PCR analyses for the *o2* gene-specific marker umc1066 (62R: ACACTCTGGCCCATGAACAC, 66R: ACAGCAATACAGGGGACCAG) and other background SSR markers were carried out in a 10 μl reaction containing 2 μl of 20 ng template DNA, 2 mM of MgCl_2_, 1 mM of dNTPs, 2 μM of primer pair, and 1.5U of Taq polymerase. The template DNA underwent an initial denaturation at 94 °C for 7 min, followed by 35 cycles of denaturation at 94 °C for 30 s, annealing at 60 °C for 30 s and extension at 72 °C for 45 s followed by a final extension at 72 °C for 7 min. The amplified PCR products were run using a 3% agarose gel for 3 h with the addition of 5 µl bromophenol blue, and the resolution was documented after 3 h.

### Marker aided transfer of *crtRB1* and *o2* genes

**Figure 2:**
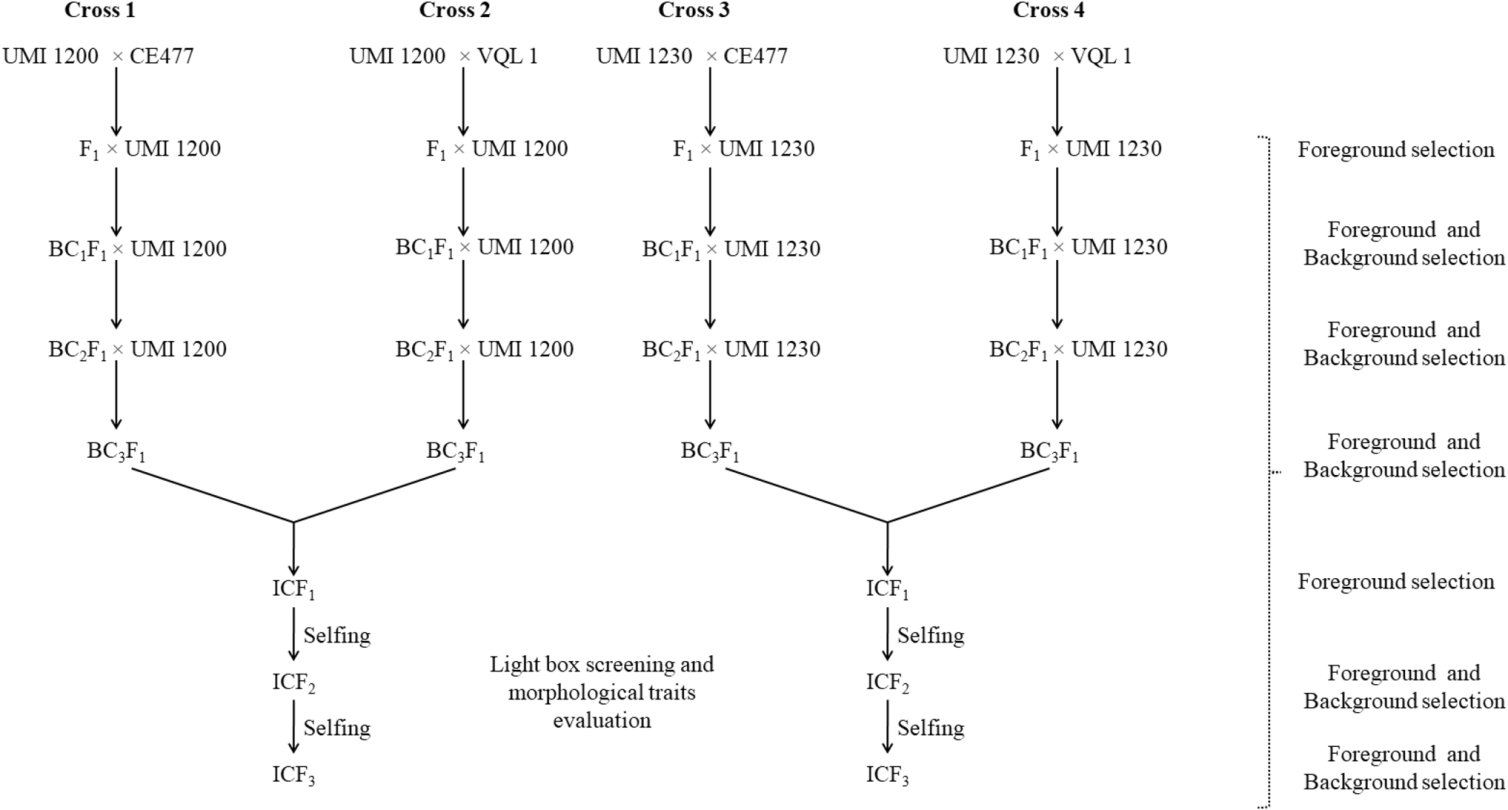
Marker assisted backcrossing scheme (MABC) used to generate the intercross (IC) population. Cross 1 (UMI 1200 × CE477); Cross 2 (UMI1200 × VQL 1); Cross 3 (UMI1230 × CE477); Cross 4 (UMI1230 × VQL 1). Crossing between parents (Kharif season, June to September 2015), F_1_ (Rabi season, November to March 2015–2016), BC_1_F_1_ (Kharif season, June to September 2016), BC_2_F_1_ (Rabi season, November to March 2016–2017), BC_3_F_1_ (Kharif season, June to September 2017), ICF_1_ (Kharif season, June to September 2018), ICF_2_ (Rabi season, November to March 2018–2019), and ICF_3_ (Kharif season, June to September 2019).

Four crossing programs, UMI1200 × CE477 (Cross-1), UMI1200 × VQL1 (Cross-2), UMI1230 × CE477 (Cross-3), and UMI1230 × VQL1 (Cross-4) were initiated to develop the nutrients rich lines using UMI1200 and UMI1230 (Recurrent) and CE477 and VQL1 (Donor) (Fig. 2). The F_1_s from all the four crosses were verified for the existence of *crtRB1* and *o2* genes in heterozygous form with foreground markers and then backcrossed with UMI1200 or UMI1230 to produce BC_1_F_1_. The BC_1_F_1_ lines having *crtRB1* (Cross-1and 3) and *o2* (Cross-2 and 4) in heterozygous form were selected with foreground markers. The foreground positives from BC_1_F_1_ were subjected to background selection to identify the plants with maximum recovery of recurrent parent genome using polymorphic SSR markers. Similarly, another two rounds of backcrossing followed by foreground and background selection generated BC_3_F_1_ lines having *crtRB1* (Cross-1 and 3) and *o2* (Cross-2 and 4) with maximum recovery of recurrent parent genome. The final lines were crossed to produce intercross F_1_s (ICF_1_) to combine the *crtRB1* and *o2* genes into a single plant. The heterozygous form in ICF_1_ was confirmed by foreground markers and then selfed to two generations to produce ICF_3._ The ICF_2_ and ICF_3_ generations were subjected to the foreground and background selection.

### Observation of kernel modification via lightbox screening

The *o2o2* allele that is associated with the increased lysine and tryptophan content is also associated with an undesirable character of kernel softness that can be visualized as opaqueness in the kernels. Based on the opaqueness, the kernels can be categorized into five levels: 0%, 25%, 50%, 75%, and 100%. Usually, 25% and 50% kernels are selected since they are certain to contain the *o2o2* gene in a homozygous recessive state. Whereas, the other categories contain the *o2* gene in either heterozygous or homozygous dominant condition and are heavily susceptible to unfavourable irregularities. A lightbox apparatus is used to differentiate the level of kernel opaqueness as an indirect measure of the kernel softness. Hence, by the dual selection technique of lightbox screening and foreground selection, the *o2o2* allele is guaranteed in the population. The ICF_2_ and ICF_3_ generation lines were subjected to the lightbox screening and the lines exhibiting 25% opaqueness are selected to fix the *o2* allele in the homozygous recessive state.

### Characterization of ICF_3_ lines for morphological traits

The newly developed intercross lines from both the cross combinations were planted along with the donor and recurrent parent. The plants were maintained with a distance of 20 cm, row spacing of 60 cm, and a row length of 3 m. Good agronomic practices were maintained during the growing period of the crop. Randomized Block Design (RBD) was performed with three replication. Randomly five plants were selected for the morphological trait evaluation. The recovery percentage of the recurrent parents was calculated according to the previous researchers^29,33^. The plants were examined for the agronomic performance by measuring 14 morphological characters viz., days to tasseling (DT, in days), days to silking (DS, in days), plant height (PH, cm), ear height (EH, cm), tassel length (TL, cm), number of tassel branches (NTB). leaf length (LL, cm), leaf breadth (LB, cm), ear length (EL, cm), number of kernels rows per ear (NKRE), number of kernels per row (NKR), ear weight (EW, g), 100 kernel weight (KW, g) and single plant yield (SPY, g). All the characterizations were done according to the descriptors suggested by the International Board for Plant Genetic Resources^43^.

### Analysis of β carotene, lysine, and tryptophan

The kernels from the ICF_3_ generation were examined for β-carotene, lysine, and tryptophan. The extraction of β-carotene was done following the method given by Kurilich and Juvik^44^ and measured with the help of High-Performance Liquid Chromatography (HPLC). The final samples were eluted in a C30 column using a mobile phase consisting of acetonitrile: dichloromethane: methanol in the ratio of 75:20:5, and the flow rate was found to be 0.4 ml/min. The standard curve was constructed based on three different dilutions (1, 10, and 100 ppm) of standard beta carotene obtained from M/s Sigma Aldrich, USA. The lysine and tryptophan contents were measured following the colorimetric method^45^. The samples were measured using the spectrophotometer at a wavelength of 390 nm for lysine and 560 nm for tryptophan, and the levels were expressed in percent^46^.

### Statement for the use of plant materials

The study complies with local and national regulations.

## Supplementary Information

Supplementary Figure 1.

Supplementary Tables.

## Abbreviations

EL: Ear length
EW: Ear weight
DS: Days to silking
DT: Days to tasseling
EH: Ear height
HPLC: High performance liquid chromatography
KW: Kernel weight
LB: Leaf breadth
LL: Leaf length
MABB: Marker assisted backcross breeding
MAGP: Marker assisted gene pyramiding
NKR: Number of kernel rows
NKRE: Number of kernel rows per ear
NTB: Number of tassel branches
PH: Plant height
PCR: Polymerase chain reaction
PME: Protein energy malnutrition
RPGR: Recurrent parent genome recovery
SPY: Single plant yield
SSR: Simple sequence repeat
TL: Tassel length
UNICEF: United Nations International Childrens Emergency Fund
USA: United States of America
VAD: Vitamin A deficiency
