# Supplementary material for: Development of β-carotene, lysine, and tryptophan-rich maize (Zea mays) inbreds through marker-assisted gene pyramiding
Source: Sci Rep. 2022 May 20;12:8551. doi: 10.1038/s41598-022-11585-y (PMC9123160; doi:10.1038/s41598-022-11585-y)
Supplement: Supplementary file 1 — Supplementary Figure 1. [file 41598_2022_11585_MOESM1_ESM.pdf]

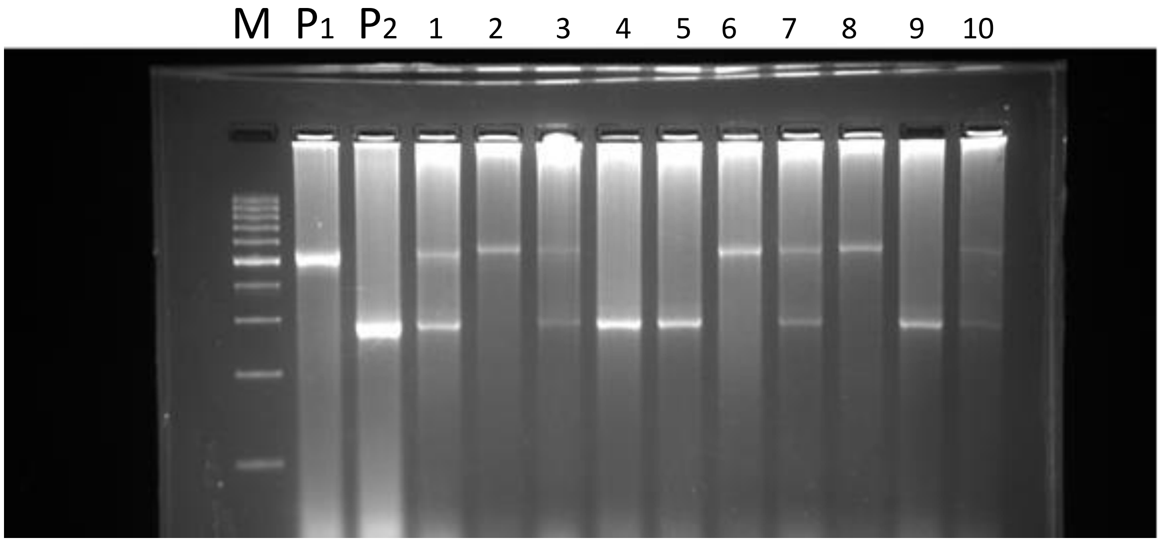

Figure 1. Foreground and background selection and morphological traits evaluation in ICF2 and ICF3 populations. (a), Foreground selection of ICF_2 lines from DBT-IC-$\beta_1\sigma_4$ using *crtRB1* gene specific marker *crtRB1* 3'TE, (M) Ladder (100 bp), (P_1) CE477, (P_2) UMI1200, (1-10) ICF_2 plants

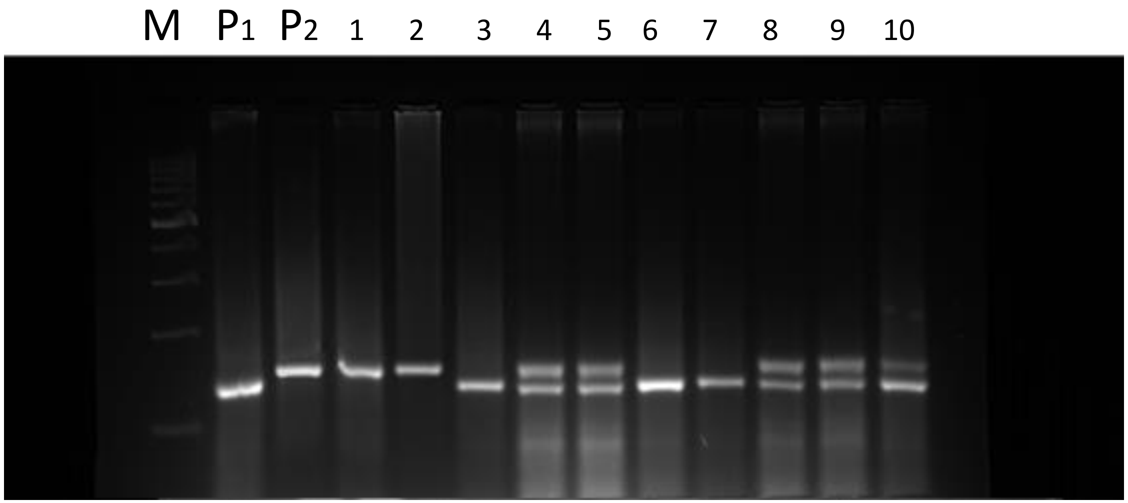

Figure 1. Foreground and background selection and morphological traits evaluation in ICF2 and ICF3 populations. (b), Foreground selection of ICF_2 lines from DBT-IC-$\beta_1\sigma_4$, using *o2* gene linked marker umc1066, (M) Ladder (100 bp), (P_1) UMI1200, (P_2) VQL 1, (1-10) ICF_2 plants

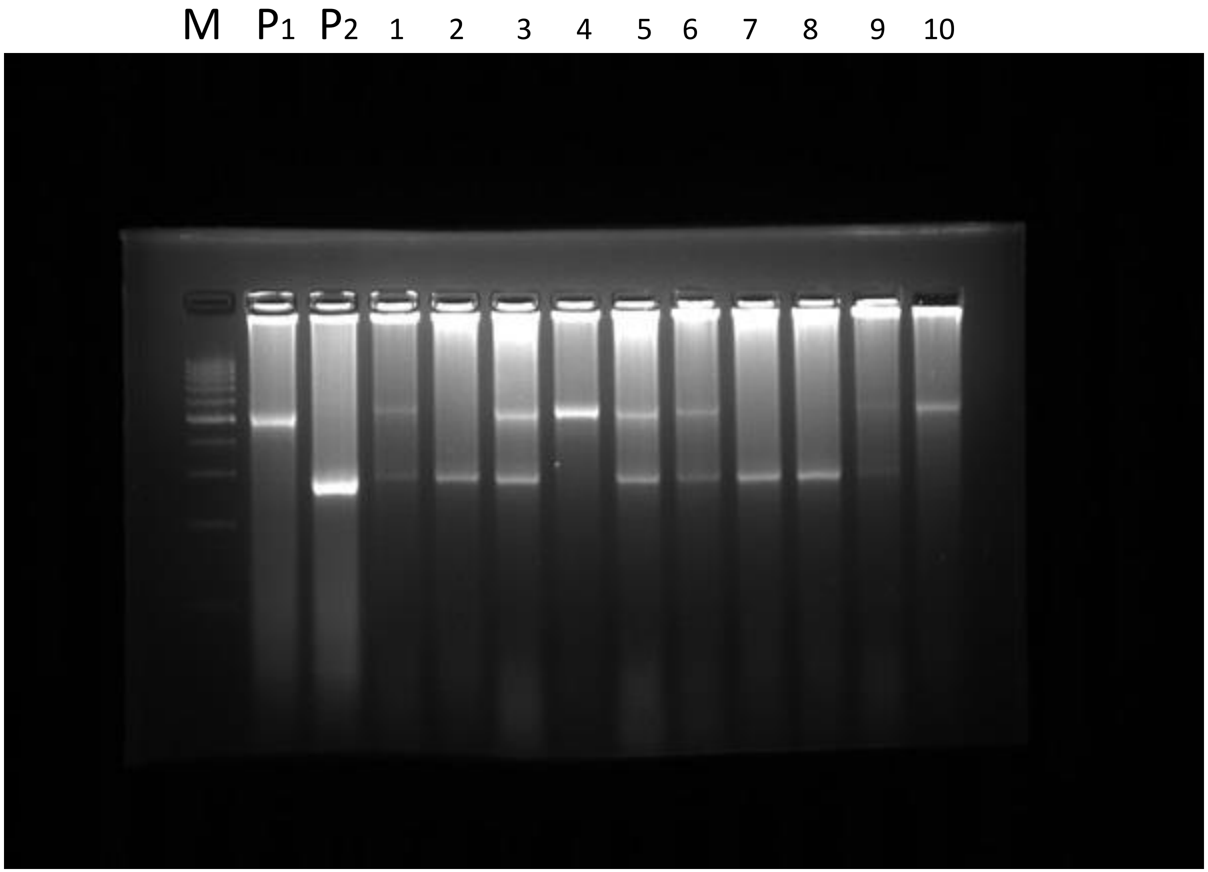

Figure 1. Foreground and background selection and morphological traits evaluation in ICF2 and ICF3 populations. (c), Foreground selection of ICF_2 lines from DBT-IC-$\beta_2\sigma_5$ using *crtRB1* gene specific marker *crtRB1* 3’TE, (M) Ladder (100 bp), (P_1) CE477, (P_2) UMI1230, (1-10) ICF_2 plants

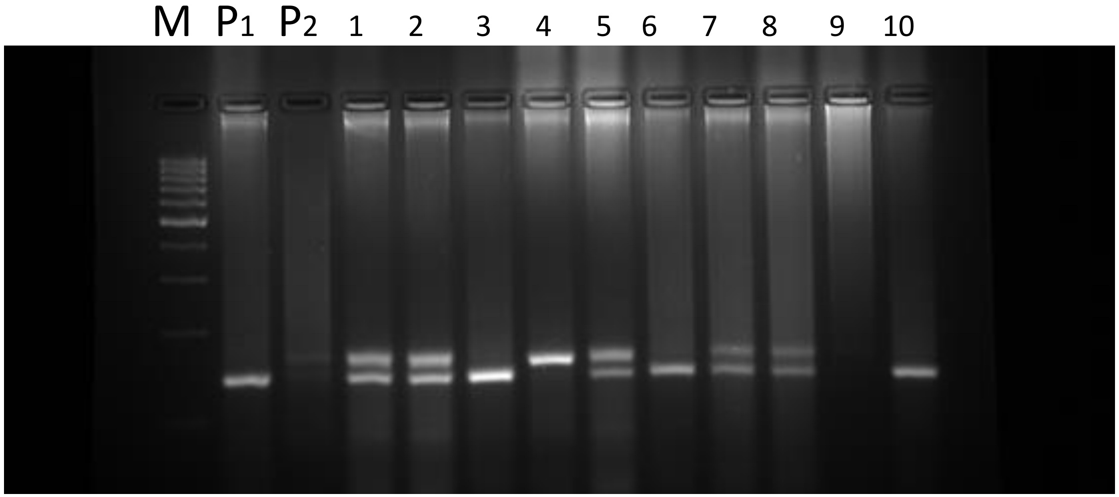

Figure 1. Foreground and background selection and morphological traits evaluation in ICF 2 and ICF3 populations. (d), Foreground selection of ICF_2 lines from DBT-IC-$\beta_2\sigma_5$ using *o2* gene linked marker umc1066, (M) Ladder (100 bp), (P_1) UMI1230, (P_2) VQL 1, (1-10) ICF_2 plants

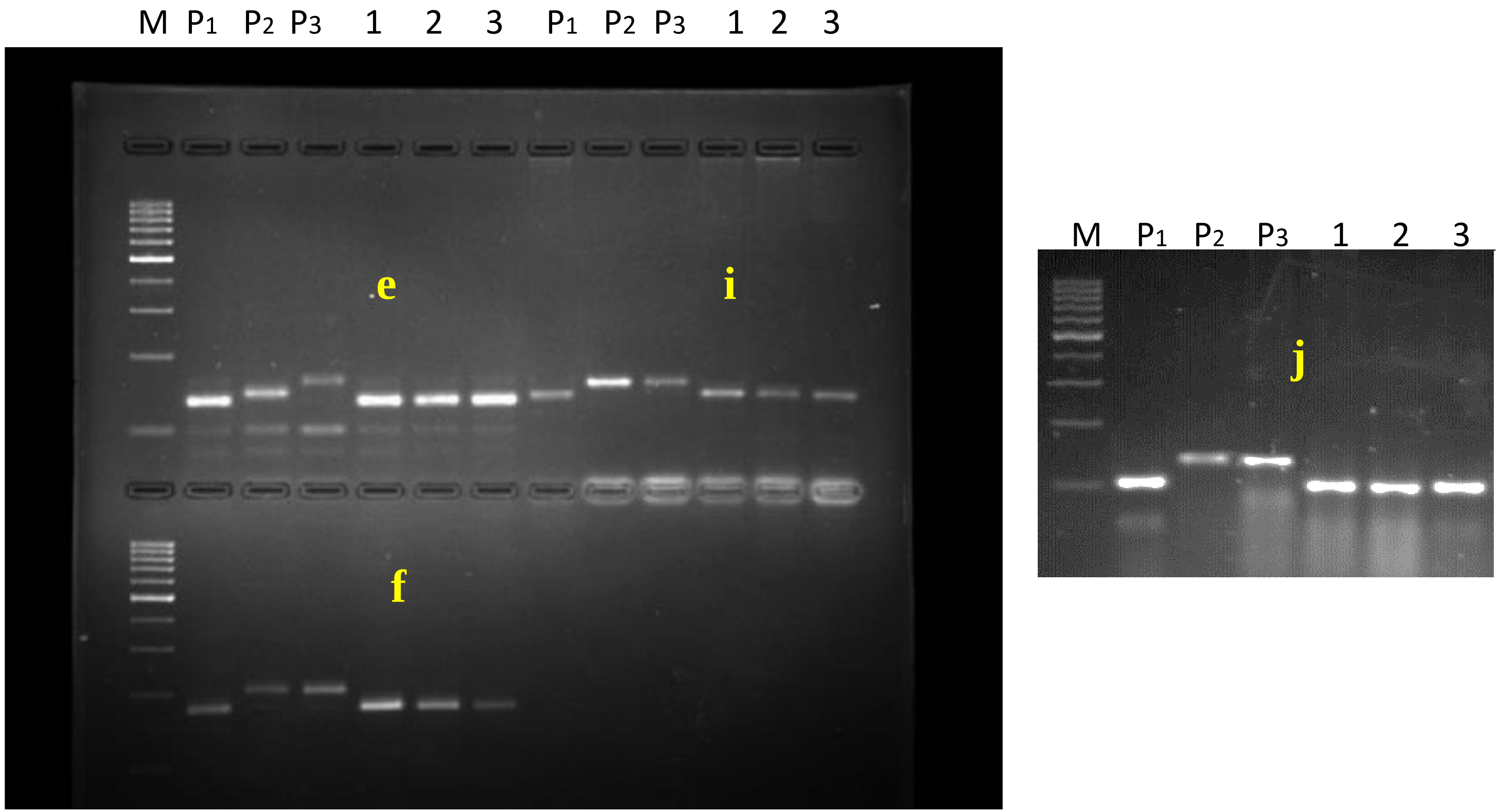

Figure 1. Foreground and background selection and morphological traits evaluation in ICF2 and ICF3 populations. (e,f, i and j), Background selection of ICF_3 lines from DBT-IC-$\beta_1\sigma_4$, (M) Ladder (100 bp), (P_1) UMI1200, (P_2) CE477, (P_3) VQL 1, (1-3) ICF_3 plants

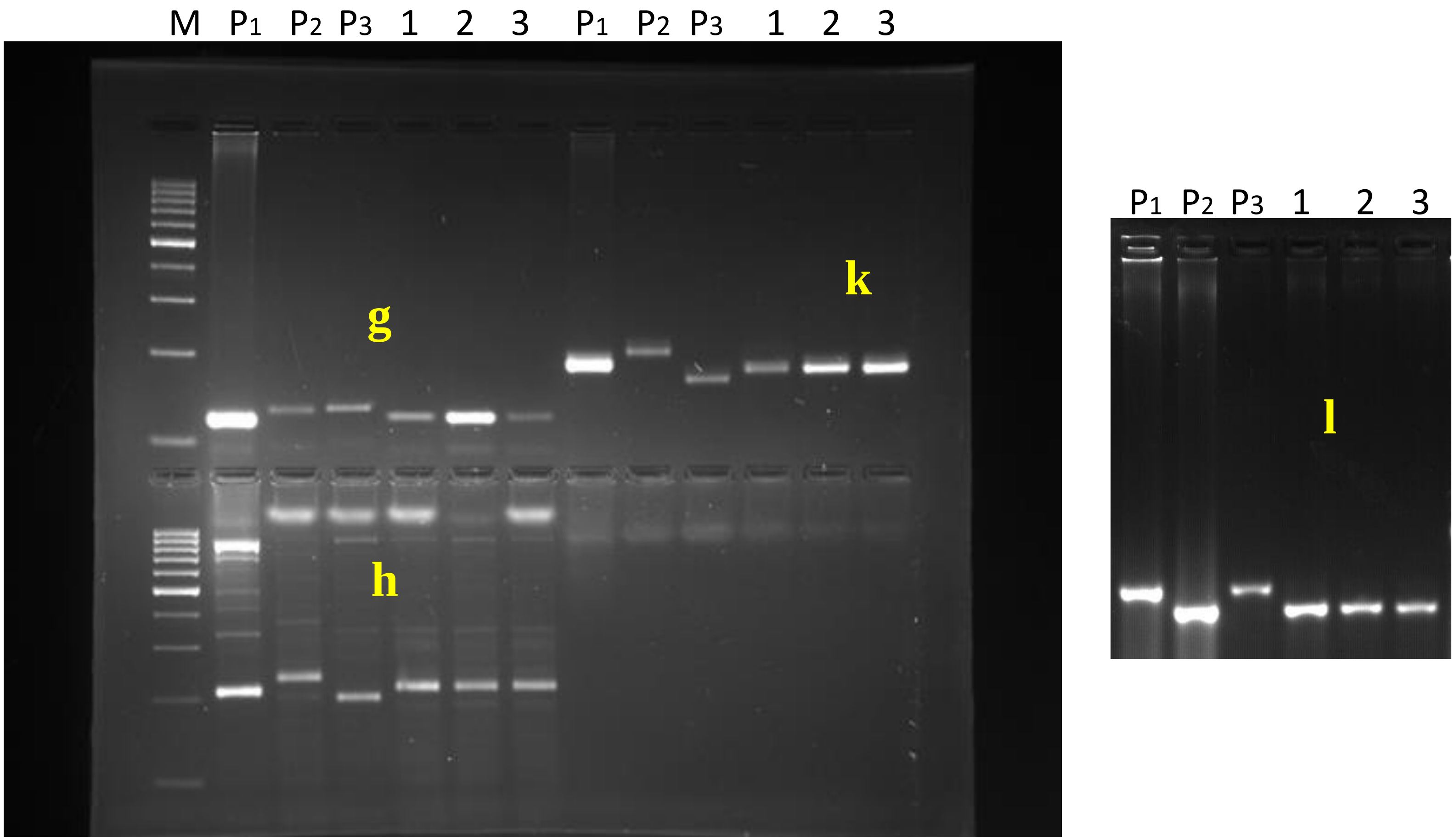

Figure 1. Foreground and background selection and morphological traits evaluation in ICF2 and ICF3 populations. (g,h,k, and l), Background selection of ICF_3 lines from DBT-IC-$\beta_2\sigma_5$, (M) Ladder (100 bp), (P_1) UMI1230, (P_2) CE477, (P_3) VQL 1, (1-3) ICF_2 plants.
