# Supplementary material for: Development of β-carotene, lysine, and tryptophan-rich maize (Zea mays) inbreds through marker-assisted gene pyramiding
Source: Sci Rep. 2022 May 20;12:8551. doi: 10.1038/s41598-022-11585-y (PMC9123160; doi:10.1038/s41598-022-11585-y)
Supplement: Supplementary file 2 — Supplementary Tables. [file 41598_2022_11585_MOESM2_ESM.doc]

| **Populations** | **Generations** | **No of polymorphic markers** | **Range (%)** | **Average (%)** |
| --- | --- | --- | --- | --- |
| UMI1200 × CE477 (Cross I) | BC_1_F_1_ | 112 | 52.82-56.41 | 54.84 |
|  | BC_2_F_1_ |  | 76.28-84.19 | 81.37 |
|  | BC_3_F_1_ |  | 86.35-88.52 | 87.74 |
| UMI1230 × CE477 (Cross III) | BC_1_F_1_ | 114 | 53.87-57.69 | 55.12 |
|  | BC_2_F_1_ |  | 78.37-86.54 | 83.62 |
|  | BC_3_F_1_ |  | 86.75-88.84 | 87.84 |

**Supplementary Table S1. Recovery percentage of the recurrent parent genome for the cross I and III**

| **Populations** | **Generations** | **No of polymorphic markers** | **Range (%)** | **Average (%)** |
| --- | --- | --- | --- | --- |
| UMI1200 × VQL 1 (Cross II) | BC_1_F_1_ | 106 | 62.13-74.25 | 69.38 |
|  | BC_2_F_1_ |  | 82.42-85.61 | 84.16 |
|  | BC_3_F_1_ |  | 86.14-88.21 | 87.45 |
| UMI1230 × VQL 1 (Cross IV) | BC_1_F_1_ | 90 | 68.60-76.20 | 72.70 |
|  | BC_2_F_1_ |  | 81.24-85.13 | 83.27 |
|  | BC_3_F_1_ |  | 87.56-89.42 | 88.41 |

**Supplementary Table S2. Recovery percentage of the recurrent parent genome for the cross II and IV**

| **Populations** | **Generations** | **No of polymorphic markers** | **Range (%)** | **Average (%)** |
| --- | --- | --- | --- | --- |
| DBT-IC-β_1_σ_4_ | ICF_1_ | 66 | 87.28-90.38 | 88.62 |
|  | ICF_2_ |  | 87.82-90.42 | 89.58 |
|  | ICF_3_ |  | 89.54-90.62 | 90.21 |
| DBT-IC-β_2_σ_5_ | ICF_1_ | 82 | 87.06-90.87 | 88.75 |
|  | ICF_2_ |  | 87.58-90.97 | 89.49 |
|  | ICF_3_ |  | 88.14-91.71 | 89.63 |

**Supplementary Table S3.** Recovery percentage of the recurrent parent genome for the Intercross populationss
